# Three-Dimensional Internal Voids and Marginal Adaptation in Deep Margin Elevation Technique: Efficiency of Highly Filled Flowable Composites

**DOI:** 10.3290/j.jad.b5759489

**Published:** 2024-10-14

**Authors:** Allegra Baldi, Tommaso Rossi, Allegra Comba, Luca Monticone, Gaetano Paolone, Isabella Sannino, Alessandro Vichi, Cecilia Goracci, Nicola Scotti

**Affiliations:** a PhD Student, Department of Surgical Sciences, Dental School, University of Turin, Turin, Italy. Manuscript writer.; b PhD Student, Department of Surgical Sciences, Dental School, University of Turin, Turin, Italy. Sample preparation and test.; c Assistant Professor, Department of Surgical Sciences, Dental School, University of Turin, Turin, Italy. Data analysis, manuscript writer.; d Clinical Tutor, Department of Surgical Sciences, Dental School, University of Turin, Turin, Italy. Sample preparation and test.; e PhD Student, Department of Dentistry, IRCCS San Raffaele Hospital and Dental School, Vita Salute University, 20158 Milan, Italy. Manuscript revision.; f PhD Student, Department of Applied Science and Technology, Politecnico di Torino, Turin, Italy. Data analysis, additional sample preparation after first revision.; g Senior Lecturer, Dental Academy, University of Portsmouth, William Beatty Building, Hampshire Terrace, Portsmouth, UK. Manuscript revision.; h Associate Professor, Department of Medical Biotechnologies, University of Siena, 53100 Siena, Italy. Statistical analysis.; i Associate Professor, Department of Surgical Sciences, Dental School, University of Turin, Turin, Italy. Coordinator of the research project.

**Keywords:** 3D interfacial gap, adhesive interface, chewing simulator, highly filled flowable composites, internal voids, mechanical aging, micro-CT

## Abstract

**Purpose:**

To evaluate interfacial three-dimensional adaptation and internal voids of different flowable materials before and after cyclic fatigue in a simulated deep-margin elevation scenario.

**Methods:**

Eighty (n = 80) extracted premolars were selected and two Class II cavities were prepared. The mesial one with cervical margin 1 mm above the cementum-enamel junction (CEJ) and the distal one with cervical margin 1 mm below the CEJ. After performing adhesive procedures, specimens were divided into four groups according to the employed materials for 2 mm horizontal deep-margin relocation: nanohybrid composite (Clearfil ES2, Kuraray); conventional viscosity flowable composite (Tetric Flow, Ivoclar); medium viscosity flowable composite (Majesty ES2 Low Flow, Kuraray); high viscosity flowable composite (Majesty ES2 Super Low Flow, Kuraray). All restorations were finalized by oblique layering with nanohybrid composite (Clearfil ES2, Kuraray). To reveal interfacial and internal gap progression, specimens were scanned with a micro-CT (SkyScan 1172), before and after 500,000 cycles of mechanical chewing simulation (50 N, 1 Hz). Data were imported into Mimics software after smoothing and region growing. Only the 2 mm margin relocation volumes were considered. Obtained masks were analyzed for noise removal and volume calculation.

At baseline, interfacial gap progression and internal voids, expressed in mm^3^, were collected and statistically analyzed with two-way ANOVA (α < 0.05) for the variables substrate and restorative materials followed by Tukey post-hoc test. An additional two-way ANOVA test, followed by Tukey post-hoc test, was performed to evaluate variation in interfacial gap progression after mechanical aging.

**Results:**

At baseline, the ANOVA test showed a significant difference for the variable restorative materials (p = 0.01). More specifically, the Tukey post-hoc test revealed that the highly filled medium viscosity composite performed better than the conventional viscosity composite at baseline for the interfacial gap. The internal voids ANOVA test at baseline reported no significant differences for the variable tested.

Analysis of variance for internal gap progression after thermocycling showed no differences for both substrate and restorative material employed.

**Conclusions:**

Highly filled medium viscosity composite performed significantly better than the conventional viscosity flowable composite for what concern baseline interfacial gaps. Artificial aging with a chewing simulator and thermocycling did not affect interfacial gap progression on enamel and dentin. The tested restorative materials performed equally after aging.

Deep margin elevation (DME) is a consolidated technique that helps avoid crown lengthening surgery when the supracrestal connective attachment is not violated, a situation that typically occurs in deep Class II cavities.^16,22,47^ The management of adhesive procedures in DME is complicated by the difficulty to maintain isolation and the small quantity of available enamel in the cervical margin, which makes adhesion less reliable.^14,41,50^

The original technique to perform DME is the so-called open-sandwich protocol, which consists of layering a horizontal increment of a different, easier-to-manipulate material at the base of the cavity before finalizing the restoration with a packable composite. The original DME protocol used resin-modified glass-ionomer, but more recently resin-based composites (RBCs) replaced glass-ionomer material due to their good mechanical properties, easier manipulation, and adaptation to deep cavities.^7,18,49^ However, RBCs are sensitive to shrinkage stress during their polymerization, which can result in debonding and interfacial gaps^1,43^ that can lead to restorative failures.^23^ Moreover, defects such as voids and bubbles, which are generated by air entrapment within the materials, have been demonstrated to be harmful to the mechanical characteristics of RBC, especially under fatigue loading.^40^ In fact, fractography researches have clearly shown that voids are defects that might initiate cracks.^8^ In addition, stress concentration around voids is also believed to reduce fatigue resistance,^17^ increase wear, and ultimately lower restoration longevity and performance.^39^

Among RBCs, flowable RBCs traditionally possess the characteristic of having low viscosity because of their lower quantity of inorganic fillers (37–53%) compared to packable RBCs (50–70%).^7^ Consequently, flowable RBCs contain a higher quantity of monomers and thus they present higher volumetric shrinkage while curing and lower physical proprieties. On the other hand, lower filler content results in greater internal absorption of polymerization stresses, ultimately leading to higher flexibility and elasticity, with reduced stress development on the adhesive interface.^11,28^ Moreover, the improved wetting simplifies clinical adaptation and allows a closer contact with the cervical margin, which is critical in DME.^12^

The above-mentioned properties could reduce interfacial gap formation during the curing phase, to the point that some authors proposed them as liners under nanohybrid RBCs.^42,53^ This topic is still debated, since some authors have shown that there is no evidence of the improvement of marginal adaptation placing a first layer of flowable composite in Class II restorations.^33^ On the other side, other studies reported reduced microleakage and improved marginal integrity when flowable RBCs were applied as cavity liners,^15,29^ under nanohybrid and ormocer composites,^26^ especially on cervical margins.^32^ Another key point that has yet to be clarified is the flowable RBCs performance when subjected to cyclic loads, that are well-known to have a deleterious effect on all adhesive interfaces.^48^ In fact, the low elasticity modulus makes flowable composites more susceptible to elastic deformation under chewing loads,^3^ raising concerns about their long-term performances when subjected to fatigue. In fact, due to the lower percentage of filler load, flowable composites generally show lower mechanical and physical properties compared with traditional nanohybrid RBCs.^21^

In order to compensate for this problem, highly filled flowable RBCs (HFRBCs) have been recently introduced in commerce, but there is still a lack of evidence about their interfacial behavior. HFRBCs seem clinically promising, with comparable effectiveness, according to FDI criteria, compared to conventional RBCs.^25^ Thanks to their higher filler content these flowable composites should be subjected to lower volumetric shrinkage during polymerization and possess higher mechanical properties compared to conventional flowable RBCs. However, a recent study by Sagsoz et al showed that bond strength values of two HFRBCs were not superior to other composites,^45^ raising doubts about their interfacial stress-development behavior.

To the best of the authors’ knowledge, there are no studies that analyzed the interfacial and internal behavior, before and after cyclic fatigue, of HFRBCs. Thus, the objective of the present study was to evaluate, through micro-CT 3D analysis, marginal adaptation, and internal voids of two HFRBCs applied to a DME scenario. The null hypotheses tested were that 3D interfacial adaptation and internal voids during the DME technique would not influence (1) the different tested RBCs and (2) the cyclic fatigue simulation.

## Materials and Methods

Eighty (n = 80) intact human upper premolars, extracted for periodontal reasons within four months, were selected and stored in water. Sample size calculation was performed based on previous studies using the G*Power 3.1 software (Heinrich Heine University Dusseldorf). After calculus cleaning and debridement with an ultrasonic device, the following inclusion criteria were applied: no carious lesions, demineralization, or visible cracks under 10× optical magnification and transillumination. A trained operator performed two Class II cavities preparations on each tooth with a flat head diamond bur (cod. 836; Komet), with the following standardized parameters: 3 mm in buccal-lingual direction, 1.5 mm in mesio-distal direction; mesial box had an enamel cervical margin 1 mm above the cementum-enamel junction (CEJ), while the distal box had a dentin-cervical margin 1 mm below the CEJ. According to cavity design, in order to keep the focus on the substrate, the term “enamel restoration” will be thereby used for the mesial restoration and “dentin restoration” for the distal restoration.

Each linear measurement was carefully controlled with a periodontal probe by a second expert operator. A circumferential steel matrix was applied (Automatrix, Dentsply) and tightened until a perfect fit with the cervical margin was achieved.

The following adhesive procedures were performed for all samples: 30 s selective enamel etching with 35% phosphoric acid (K-etchant, Kuraray Noritake Dental), 30 s rinse, and 30 s air-dry. A two-step self-etch adhesive system was then applied following the manufacturer’s instructions (Clearfil SE Bond 2, Kuraray Noritake) and light-cured for 40 s at 1400 mW/cm2 with a LED lamp (Cefalux 2, VOCO).

After that, specimens were divided into four groups (n = 20) according to the employed materials and a single experienced operator performed the restorations:

- G1: Clearfil ES 2 (Kuraray Noritake) was applied with an initial 2 mm-thick horizontal layer. The restoration was then finalized by oblique layering with the same material.
- G2: Tetric Flow (Ivoclar) was applied in a 2 mm-thick horizontal layer. A 10 s setting time was waited before light-curing in order to achieve optimal adaptation. The restoration was then finalized by oblique layering with a conventional paste composite (Clearfil ES 2, Kuraray Noritake).
- G3: Majesty ES Low Flow (Kuraray Noritake) was applied in a 2-mm-thick horizontal layer. A 10 s setting time was waited before light-curing in order to achieve optimal adaptation. The restoration was then finalized by oblique layering with a conventional paste composite (Clearfil ES 2, Kuraray Noritake).
- G4: Majesty ES Super Low Flow (Kuraray Noritake) was applied in a 2 mm-thick horizontal layer. A 10 s setting time was waited before light-curing in order to achieve optimal adaptation. The restoration was then finalized by horizontal layering with a conventional paste composite (Clearfil ES 2, Kuraray Noritake).

**Fig 1:**
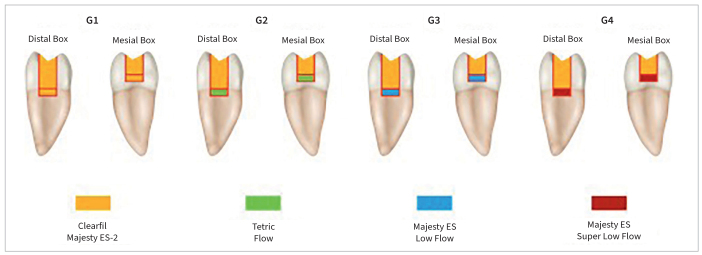
Scheme of four tested groups and different materials used.

A scheme of tested scenarios is reported in Figure 1, while a summary of the employed materials, alongside their classification, main chemical components and main mechanical properties, is given in Table 1.

**Table 1:** Summary of used materials, alongside classification, main components and main mechanical properties

Material	Classification	Main Components	Mechanical Properties
Clearfil Majesty ES-2, Kuraray Noritake	Nanohybrid composite	Resin matrix: Bis-GMA, hydrophobic aromatic DMA and hydrophobic aliphatic, dl-camphorquinone Inorganic filler: silanated barium glass (particle size 0.37–1.5 µm) and prepolymerized organic filler. 78 wt%, 40 vol%*	Flexural Strenght: 118 MPa Compressive Strenght: 347 MPa*
Tetric Flow, Ivoclar	Nano-hybrid flowable resin composite	Resin matrix: Bis-GMA, UDMA, D3MA Inorganic filler: barium glass, ytterbium fluoride, silica (particle size 0.04–3 µm) 57.5 wt%, 30.7 vol%*	Flexural Strenght: 114 MPa Compressive Strenght: 260 MPa*
Majesty ES Low Flow, Kuraray Noritake	Highly-filled flowable resin composite – medium viscosity	Resin matrix: TEGDMA, hydrophobic aromatic DMA, dl-camphorquinone, PI Inorganic filler: barium glass filler (particle size 3 µm), silica filler (particle size 20 nm), 81 wt%, 62 vol% (49)	Flexural Strenght: 151 MPa Compressive Strenght: 373 MPa*
Majesty ES Super Low Flow, Kuraray Noritake	Highly-filled flowable resin composite – high viscosity	Resin matrix: TEGDMA, hydrophobic aromatic DMA, dl-camphorquinone, PI Inorganic filler: barium glass filler, silica filler 78 wt%, 64 vol% (6)	Flexural Strength: 152 MPa Compressive Strength: 374 MPa*
Clearfil SE Bond 2, Kuraray Noritake	Self-etch two-steps adhesive	Primer: 10-Methacryloyloxydecyl dihydrogen phosphate (MDP), 2-ydroxyethyl methacrylate (HEMA), Hydrophilic aliphatic dimetacrylate, dl-Camphoroquinone, water Bonding: 10-Methacryloyloxydecyl dihydrogen phosphate (MDP), 2-ydroxyethyl methacrylate (HEMA), Bisphenol A diglycidylmethacrylate (Bis-GMA), Hydrophilic aliphatic dimetacrylate, dl-Camphoroquinone, initiators, accelerators, silanated colloidal silica*	

The superscript asterisk indicate that the reported information was given directly by the material’s producer.

All layers were light-cured for 40 s with an LED lamp (Cefalux 2, VOCO). A final 20 s light-curing cycle was performed under air-barrier transparent gel. Samples were polished by an expert operator using fine and extra-fine diamond burs, rubber points (Twist DIA, Kuraray Noritake), and nylon brush. A second expert operator confirmed the clinical acceptability of the obtained restorations.

Marginal adaptation and internal voids of each restoration were evaluated using micro-CT (SkyScan 1172 Micro-CT, Bruker). Acquisition was performed with the following parameters: voltage = 100 kV; current = 100 µA; aluminum and copper (Al+Cu) filter; pixel size = 14.8 µm; averaging = 5; rotation step = 0.6 degrees; random movement = 10; scanning 360 degrees = OFF, total scan duration = 44 min. Images were reconstructed through NRecon software (Bruker) in order to obtain DICOM files, with the following standardized parameters: beam hardening correction = 20%, smoothing = 4; ring artifact reduction = 9. All samples were scanned and reconstructed with the exact same procedure to achieve consistency in greyscale values, fundamental in further analysis.

**Fig 2:**
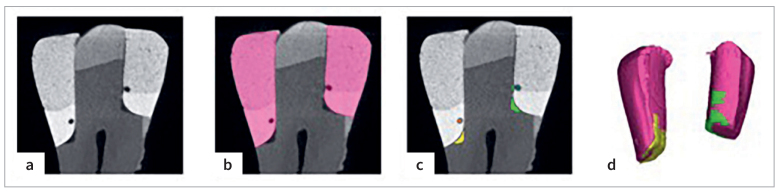
Example of software segmentation on Mimics.* (a)* Whole sample without any mask. *(b)* Pink mask represents the restoration. *(c)* The yellow mask represents the marginal adaptation volume on dentin restoration; the green mask represents the marginal adaptation volume on enamel restoration; the orange mask represents the internal void volume on dentin restoration; the blue mask represents the internal void volume on enamel restoration. *(d)* 3D rendering of restoration and marginal adaptation volumes.

The so-obtained DICOM files were processed with a segmentation software (Mimics Medical 24.0, Materialise) in order to measure three-dimensionally interfacial adaptation and internal voids/bubbles in the first two cervical millimeters of the restoration, that represent the relocation area. First of all, all parts of the restored tooth (enamel, dentin, restorative material, void areas), each one corresponding to a specific spike in the Hounsfield Unit graph, were segmented by the software in different masks, using the same Hu values (1024 to 950) for all samples to ensure consistency among data. In order to eliminate artifacts and noise, and isolate the relevant regions of interest, a standardized protocol consisting of Boolean (intersection, subtraction) and morphology (erode, close, dilate) operations was used. In order to specifically select the relocated material, a “crop volume” function was used in the z-axis, setting its height = 2 mm from the base of the cavity for all samples. Apart from the focus on the relocation, the crop step is necessary due to cavity geometry: if the 3D analysis is performed along the whole restoration, the dentin cavity will be more extended in the apico-coronal sense, therefore possessing a wider interfacial surface and making impossible a direct comparison between dentin and enamel substrates. After the selection of the voxels of interest, a check was performed through a 3D rendering preview and the volume was recorded from the “mask propriety” section. Voids and defects that interested the tooth-restoration interface were included in the “marginal adaptation volume,” while completely surrounded by restorative materials defects were included in the “internal voids volume.” The crucial steps of the procedure are synthesized in Figure 2.

After the first micro-CT scan, a CS-4.4 chewing simulator (SD, Mechatronik) was used for the mechanical aging of the specimens. The roots of the specimens were covered by a 2 mm-thick layer of a silicon impression material (Express, 3M ESPE), before being embedded in a light-curing resin (Megatray, MEGADENTA) to simulate the human periodontium’s resilience. A 6-mm-diameter steatite sphere was used with the following settings: occlusal load = 50 N; frequency = 1 Hz; downward speed = 90 mm/s; sliding movement = 2 mm over the buccal triangular crest. All restored specimens were positioned to center the sphere exactly on the central fossa of the tooth and the test was performed for 500,000 cycles in distilled water at 25°C, according to several previous studies.^48^

**Fig 3:**
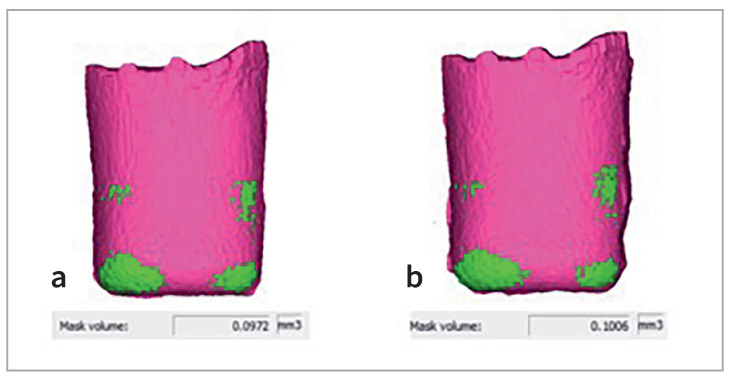
Example of data comparison before and after mechanical aging. The pink masks represent restoration, the green masks represent marginal adaptation volume. Mask volume in mm3 of marginal adaptation is obtained by “mask propriety” sectioning. *(a)* Sample before aging.* (b)* Sample after aging.

After mechanical aging, in order to reveal interfacial adaptation degradation after cyclic fatigue, specimens were subjected to a second scan, with the same protocol both in terms of acquisition and analysis. An example of data comparison, alongside the 3D rendering of marginal gaps, is reported in Figure 3.

**Fig 4:**
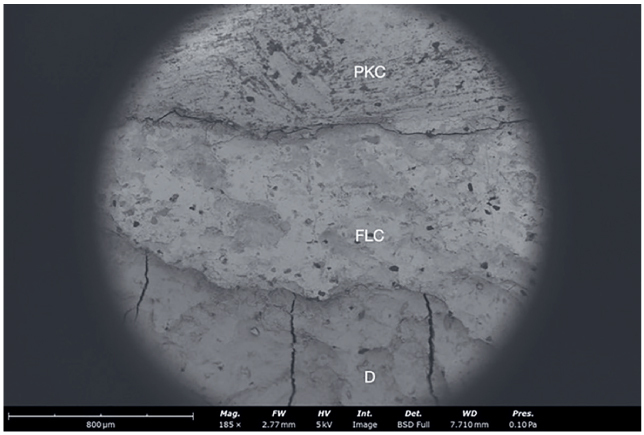
A representative SEM image of a sample with cervical margin elevation in dentin (185×). Dentin (D), high viscosity flowable composite (FLC) used for marginal relocation, and packable composite (PKC) used for the restoration are clearly visible.

**Fig 5:**
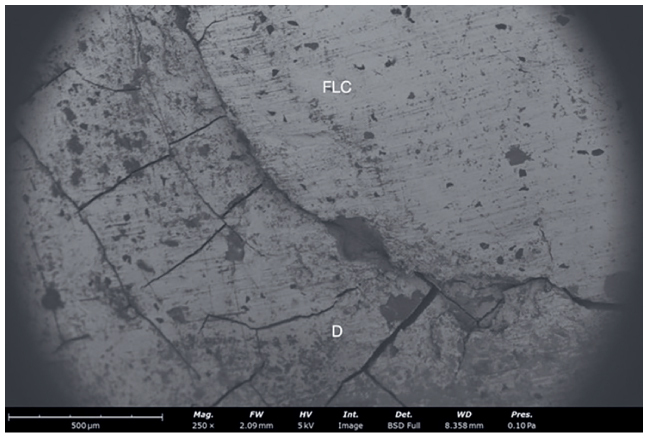
A representative SEM image of a sample with cervical margin elevation in dentin (250×). The dentin-flowable interface shows the presence of an interfacial gap. Dentin (D) and high viscosity flowable composite (FLC) used for marginal relocation are reproduced.

**Fig 6:**
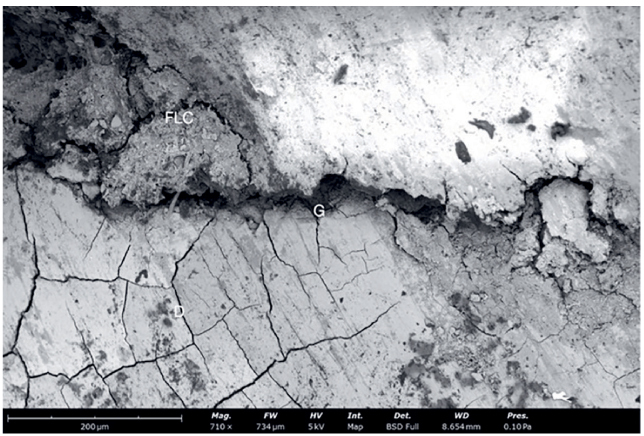
A representative SEM image of a sample with cervical margin elevation in dentin (710×). A large gap is visible at the dentin-flowable interface after aging with a chewing simulator. Dentin (D), high viscosity flowable composite (FLC) used for marginal relocation are reproduced, and gap (G).

In order to verify and investigate interfacial gaps, samples from all groups were scanned with a scanning electron microscope (SEM). Samples were dehydrated taking it through a series of increasing concentrations of a drying liquid (ethanol), ending in a 100% dehydrating liquid of the highest possible purity. The steps to achieve 100% ethanol solution were as follows: two baths of 10 min each at 10%, 20%, 30%, 50%, 70%, 90%, and three baths of 10 min each at 100%. After that specimens were platinized and scanned with SEM. Images from SEM analysis show continuity of the tooth-restoration interface (Figs 4 and 5) and defects on the marginal area (Figs 6).

At baseline, interfacial gap progression and internal voids data, expressed in mm3, were collected and statistically analyzed with two-way ANOVA (α < 0.05) for the variables substrate and restorative materials followed by Tukey post-hoc test. An additional two-way ANOVA test, followed by Tukey post-hoc test, was performed to evaluate variation in interfacial gap progression after thermocycling.

The level of significance was set at 95% (a = 0.05). The Stata software suite was utilized for all statistical analyses (Stata-Corp 14.0, College Station, TX, USA).

## Results

Average interfacial volumetric adaptation and internal voids (± SD; both expressed in mm^3^), for both enamel and dentin restorations are displayed in Table 2. Variation in interfacial gaps after chewing simulation are reported in Table 3, as a mean of the difference between after-chewing and baseline values of every single sample (± SD; expressed in mm^3^).

**Table 2:** Interfacial volumetric gap and internal voids results at baseline (before chewing simulation), both expressed as mm^3^, for all group tested

Material	Interfacial adaptation enamel restoration	Interfacial adaptation dentin restoration	Internal voids enamel restoration	Internal voids dentin restoration
G1 (Clearfil Majesty ES-2)	0.28 ± 0.24^aA^	0.29 ± 0.24^aA^	0.09 ± 0.07^aA^	0.03 ± 0.01^aA^
G2 (Tetric Flow)	0.50 ± 0.36^bA^	0.56 ± 0.53^bA^	0.01 ± 0.01^aA^	0.02 ± 0.01^aA^
G3 (Majesty ES Low Flow)	0.05 ± 0.03^aA^	0.09 ± 0.05^aA^	0.01 ± 0.01^aA^	0.05 ± 0.09^aA^
G4 (Majesty ES Super Low Flow)	0.17 ± 0.15^aA^	0.19 ± 0.16^aA^	0.05 ± 0.03^aA^	0.01 ± 0.01^aA^

Different superscript lower case letters indicate differences within the columns, different superscript upper case letters indicate differences within the rows.

**Table 3:** Interfacial volumetric gap progression after mechanical aging (chewing simulation), both expressed as mm^3^, for all groups and subgroups

Material	Interfacial adaptation enamel restoration	Interfacial adaptation dentin restoration
G1 (Clearfil Majesty ES-2)	+ 0.03 ± 0.01^aA^	+ 0.02 ± 0.01^aA^
G2 (Tetric Flow)	+ 0.02 ± 0.01^aA^	+ 0.01 ± 0.01^aA^
G3 (Majesty ES Low Flow)	+ 0.02 ± 0.01^aA^	+ 0.01 ± 0.01^aA^
G4 (Majesty ES Super Low Flow)	+ 0.02 ± 0.01^aA^	+ 0.02 ± 0.01^aA^

Different superscript lower case letters indicate differences within the columns, different superscript upper case letters indicate differences within the rows.

After ascertaining the normality (Shapiro–Wilk test) and homoscedastic (modified Levene test) assumptions of the datasets at baseline and after aging, the internal gap and volumetric voids data were analyzed with two-way analysis of variance to examine the effects of the substrate and restorative materials on the II class performed.

At baseline, two-way ANOVA test showed a significant difference for the variable restorative materials (p = 0.01). More specifically, Tukey post-hoc test revealed that the highly filled medium viscosity composite (G3 Majesty ES Low Flow) performed better than the conventional viscosity composite (G2 Tetric Flow) at baseline as concern the evaluation of interfacial gap.

The ANOVA two-way test for the evaluation of internal voids at baseline reported no significant differences for the variable tested (substrate and restorative materials).

Analysis of variance for internal gap progression after mechanical aging and thermocycling showed no differences for both substrate and restorative material employed.

## Discussion

In the present study, micro-CT 3D analysis was used to examine the interfacial and internal behavior of HFRBCs applied to DME. Both enamel and dentin substrates were tested, as well as the degradation that cyclic mechanical loading might induce.

Micro-CT was selected since it does not require sample sectioning, thus allowing multiple evaluations on the same sample and it is an excellent method to detect internal and interfacial defects of RBCs with high resolution.^35,54^ The 3D workflow was applied according to literature, in order to achieve a comprehensive analysis of the selected volume with reduced operator bias.^5^ It is important to point out that this type of analysis requires a complex post-acquisition process and a standardized protocol to follow in order to be able to compare data before and after the chewing simulation.

Based on the obtained results, the first null hypothesis was rejected since G3 performed significantly better than G2 on interfacial adaptation at baseline. Moreover, even if not significantly from a statistical point of view, G4 performed on average better than G2, confirming a good interfacial behavior of HFRBCs compared to conventional flowable materials.

The interfacial adaptation results obtained at baseline must be attributed to two main factors: the manipulability of the materials and the polymerization kinetic that develops stresses on the adhesive surface and causes interfacial gaps formation. The easier manipulability of flowable RBCs in DME is without any doubt an important clinical factor especially when dealing with dentin cervical margins.^10^ However, conventional group (G1) showed similar results in terms of adaptation compared to the other groups, meaning that this factor was not so crucial in this *in-vitro*, controlled environment. In terms of stress development, on the other hand, the amount of filler and the different monomers contained in tested materials might have led to the significant difference. In particular, both HFRBCs tested have a TEGDMA-based organic matrix, in contrast with both G1 and G2 that are Bis-GMA based. It is well-known that Bis-GMA possesses a stiff central core of phenyl ring, which makes it viscous compared with TEGDMA, which has a long, linear, and flexible structure.^31^ The higher the mobility of the chains and elasticity of the structure, the lower the stresses expressed on the interfacial area, with a consequently reduced gap onset.^52^ Moreover, filler content in volume was much higher in G3 and G4 (62% and 64%, respectively) than G2 (30.7%). As stated by Nie et al, the volume percentage of the filler is the most influential factor on volumetric shrinkage: when it decreases from 68.6% to 33.3%, volumetric shrinkage increases from 2.55% to 5.20% accordingly.^37^ Therefore, it can be assumed that HFRBCs, due to their specific formulation, are probably a good balance between filler content and monomer polymerization kinetic, with a favorable initial marginal adaptation when applied to DME while also having good manipulability compared to packable RBCs. Obtained data are also consistent with previous studies that compared posterior RBC restorations with and without flowable liner, finding no significant differences both with silver nitrate penetration and linear micro-CT analyses.^9,33^

Concerning the substrate of the cervical margin at baseline, enamel and dentin equally performed from a statistical point of view, even if dentin showed on average slightly higher values (lower adaptation). This is consistent with literature data that report equal or slightly superior interfacial performance on enamel substrate when testing microleakage.^30^ This is obviously related to the histological aspects of enamel, which is easy to dehydrate, demineralize, and infiltrate when properly pre-treated with etching.^20^ On the other hand, it is difficult to achieve optimal adhesion on dentin due to its permeability and the presence of a higher water and organic quotes.^34^ However, it is worth underlining that substrate is probably a much more critical factor in long-term clinical situations, not just because of lower bond strength, but due to biochemical and enzymatic degradation of the hybrid layer.^19^

Regarding internal voids, no significant differences were found between tested materials. This is in disagreement with a 2004 Olmez et al paper that used sample sectioning and microscopy to assess internal voids in flowable RBC used as liners in Class II restorations below CEJ.^38^ Despite the similar study design, the present study cannot be compared directly with the Olmez study, both due to the significant differences in terms of tested materials and the technique used for void assessment. As a matter of fact, sample sectioning is a semi-quantitative method that has far inferior ability to detect internal and interfacial defects compared with micro-CT, which is considered the new gold standard alongside optical coherence tomography (OCT).^46,54^ Apart from that, the “operator experience” variable plays an important role in air entrapment during layering. According to Korkmaz et al, the operator must gently apply flowable RBCs to the gingival margin in one direction with a gentle releasing action, avoiding forceful injection.^26^ In the present study, in order to minimize this bias, a single expert operator performed all the restorative procedures. In accordance with our results, it is reported in literature that differences in terms of internal voids of different RBC applied to Class II restorations decrease if the operator is experienced.^12^ This has probably led to similar results between different viscosities RBCs. Moreover, the internal void volume increases with the number of layers that are applied, but the present study only focused on the single initial layer, thus avoiding this bias.^13^

With regard to interfacial adaptation results after chewing simulation, all tested RBC showed, on average, a minor interfacial degradation (more interfacial gaps detected), without significant differences among tested materials and substrates. This is a well-known mechanism, correlated to the chewing cycle stress concentration that, in particular spots, sometimes exceeds local fracture strength, leading to crack initiation and propagation.^2^ Within the limitation of the selected aging protocol, which simulates approximately 2 years of clinical activity, all samples showed a similar behavior.^36^ Both the substrate and the material factors could gain importance with further aging and with the addition of thermal fluctuations, as demonstrated by Scotti et al in a similar protocol with double the number of loading cycles.^48^ Unsurprisingly, internal voids did not show any variation after the mechanical aging, meaning that no micro-cracks or defects developed inside tested samples in the analyzed volume.

In conclusion to data analysis of aged samples, no evidence was recorded about either the so-called stress-breaking effect of flexible liners,^24^ nor the faster degradation of RBCs with lower mechanical properties.^48^ The same conclusions were drawn by Spreafico et al in 2016, with an *in-vitro* study on DME applied to indirect ceramic restorations that showed no differences before and after loading on marginal integrity when comparing flowable and packable RBCs.^51^ Similarly, Rocca et al recorded no differences on the mechanical behavior between flowable and packable RBCs in indirect Class II restorations.^44^ This could also be explained by the similar elastic moduli of tested materials that have been shown to be important in marginal adaptation and stress distribution in direct restorations, with an ideal range from 4 to 6 GPa.^4,27^ Considering that all tested materials are within this range, it is reasonable to assume that their interfacial behavior while mechanically loaded will be similar. Accordingly, a recent finite element study on HFRBCs reported benefits in terms of shear and tensile stress distribution when materials with such elasticity were employed in DME.^6^

## Conclusions

Within the limitations of the present *in-vitro* study, it can be concluded that:

- HFRBCs showed promising results in terms of interfacial adaptation at baseline.
- Cervical substrate did not play a crucial role in relation to the tested conditions.
- No significant differences were reported in terms of internal voids among tested materials.
- Chewing simulation caused minor interfacial degradation on all tested groups, without significant differences between materials and substrates.

Further studies should confirm the obtained data and investigate the effects of longer-term mechanical and thermal aging on HFRBCs.
